# The Environment, Not Space, Dominantly Structures the Landscape Patterns of the Richness and Composition of the Tropical Understory Vegetation

**DOI:** 10.1371/journal.pone.0081308

**Published:** 2013-11-22

**Authors:** Yue-Hua Hu, Da-Yong Sheng, Yang-Zhou Xiang, Zeng-Jiang Yang, Da-Ping Xu, Ning-Nan Zhang, Lei-Lei Shi

**Affiliations:** 1 Key Laboratory of Tropical Forest Ecology, Xishuangbanna Tropical Botanical Garden, Chinese Academy of Sciences, Mengla, Yunnan, China; 2 Research Institute of Tropical Forestry, Chinese Academy of Forestry, Guangzhou, Guangdong, China; 3 South China Agricultural University, College of Forestry, Guangzhou, Guangdong, China; 4 Huizhou Institute of Forestry Science, Huizhou, Guangdong, China; 5 Guizhou Institute of Forest Inventory and Planning, Guiyang, Guizhou, China; CNRS - Université Lyon 1, France

## Abstract

The mechanisms driving the spatial patterns of species richness and composition are essential to the understanding of biodiversity. Numerous studies separately identify the contributions of the environment (niche process) and space (neutral process) to the species richness or composition at different scales, but few studies have investigated the contributions of both types of processes in the two types of data at the landscape scale. In this study, we partitioned the spatial variations in all, exotic and native understory plant species richness and composition constrained by environmental variables and space in 134 plots that were spread across 10 counties in Hainan Island in southern China. The 134 plots included 70 rubber (*Hevea brasiliensis*) plantation plots, 50 eucalyptus (*Eucalyptus urophylla*) plantation plots, and 14 secondary forest plots. RDA based variation partitioning was run to assess the contribution of environment and space to species richness and composition. The results showed that the environmental variables alone explained a large proportion of the variations in both the species richness and composition of all, native, and exotic species. The RDA results indicated that overstory composition (forest type here) plays a leading role in determining species richness and composition patterns. The alpha and beta diversities of the secondary forest plots were markedly higher than that of the two plantations. In conclusion, niche differentiation processes are the principal mechanisms that shape the alpha and beta diversities of understory plant species in Hainan Island.

## Introduction

Understanding how the number of species (richness) and species composition vary from place to place is pivotal to explaining the maintenance of biodiversity [1,2]. In the past century, many studies have investigated the mechanisms underlying the patterns of species richness [3], but the forces that determine the patterns of species composition, although key to understanding ecosystem function, conservation, and management [4], have not been systematically explored [1]. This scenario did not change until recently in response to the neutral theory raised by Hubbell [5]. Recently, increasingly rigorous quantitative studies have investigated the mechanisms that determine the species composition patterns of tree species [1,6,7], understory plant species in temperate forests [8], pteridophytes [9,10], fungi [11], herbivorous insects species [12], and fishes [13] at different scales. However, the mechanisms that govern the distributions of tropical understory plant species, which compose the majority of the floristic species diversity in the tropics [14,15], has rarely been explored.

To date, most studies have focused on the separate importance of niche and neutral factors on the species richness patterns [16–18] or the species composition patterns [6,9]. Only a few studies have explicitly explored the relative importance of both niche and neutral factors on the spatial variation in the species richness and composition. For instance, Legendre et al. [7] analyzed the contributions of topography and space to the spatial patterns in the richness and composition of tree species in a 24-ha subtropical forest plot at the local scale (

< 1 km^2^). Most of the previous studies have also been confined to single, contiguous forest plots. However, by conducting such studies at the meso and the landscape spatial scales over a spatially extensive sample of forest stands, one can incorporate additional environmental variation and evaluate how the relative importance of both niche and neutral factors change at larger spatial scales [19].

Many biotic factors are important in the determination of the spatial distributions of understory plant populations [20–23]. Among these biotic factors, Hart et al. [22] and Comita and Hubbell [24] recognized and noted the important influence of the overstory tree species composition on the species composition of the understory. Abiotic factors, such as the soil moisture [25], litter properties [20], and topography [26], are also widely used to analyze the forces that determine the understory plant species distribution pattern. Particularly at large landscape scales, the species spatial patterns respond markedly to environmental variables [27–29]. In the analysis of species spatial distributions, it is necessary to model the spatial autocorrelation effect [30–32]. The integration of the spatial autocorrelation into ecological models can help us unbiasedly understand the processes that drive the species distributions, achieve type I error control, and make more accurate prediction of the species distributions [30–33].

Because exotic species usually exert adverse influences on the primary ecosystem [34], knowledge of their habitat associations can be used to control them. In addition, the mechanisms through which exotic species diversity relates to the native species diversity is still a controversy: negative [35] and positive [36,37] relationships have been reported between the diversity patterns of exotic and native species. 

In this study, we disentangled the contributions of spatial structures and environmental variables to the richness and composition patterns of tropical understory plant species in three types of forests at the landscape scale across Hainan Island in southern China. Previous studies demonstrated that environmental heterogeneity can be significantly associated with species distributions at the meso and landscape scales [19] and that space is important to species turnover [1]. Thus, we hypothesized that environmental heterogeneity and space significantly contribute to the variations in the species richness and the species composition, respectively. In addition, Davies et al. [36] suggested that the relationship between exotic and native species is negative and positive at the small and large scales, respectively. We therefore hypothesized that the relationship between exotic and native understory plant species across Hainan Island is positive, i.e., that the numbers of native species richness and individuals increased with increases in the numbers of exotic species richness and individuals, respectively.

## Materials and Methods

### The study area

The study sites were located across Hainan Island in southern China (108°37′ ~111°05′ E, 18°10′ ~ 20°10′ N). Hainan Island is located on the northern edge of the Asian tropical rain forests and is identified as one of the 25 top-priority biodiversity hotspots for global biodiversity conservation [38]. This area experiences a tropical monsoon climate that consists of two distinct alternating wet and dry seasons. The wet season is from May to October and is mainly influenced by southeast winds from the Pacific Ocean. The rainfall in the wet season accounts for more than 80% of the annual rainfall. The dry season occurs from November to April of the following year and is dominantly affected by southwest dry winds from mainland China.

### Data collection

**Figure 1:**
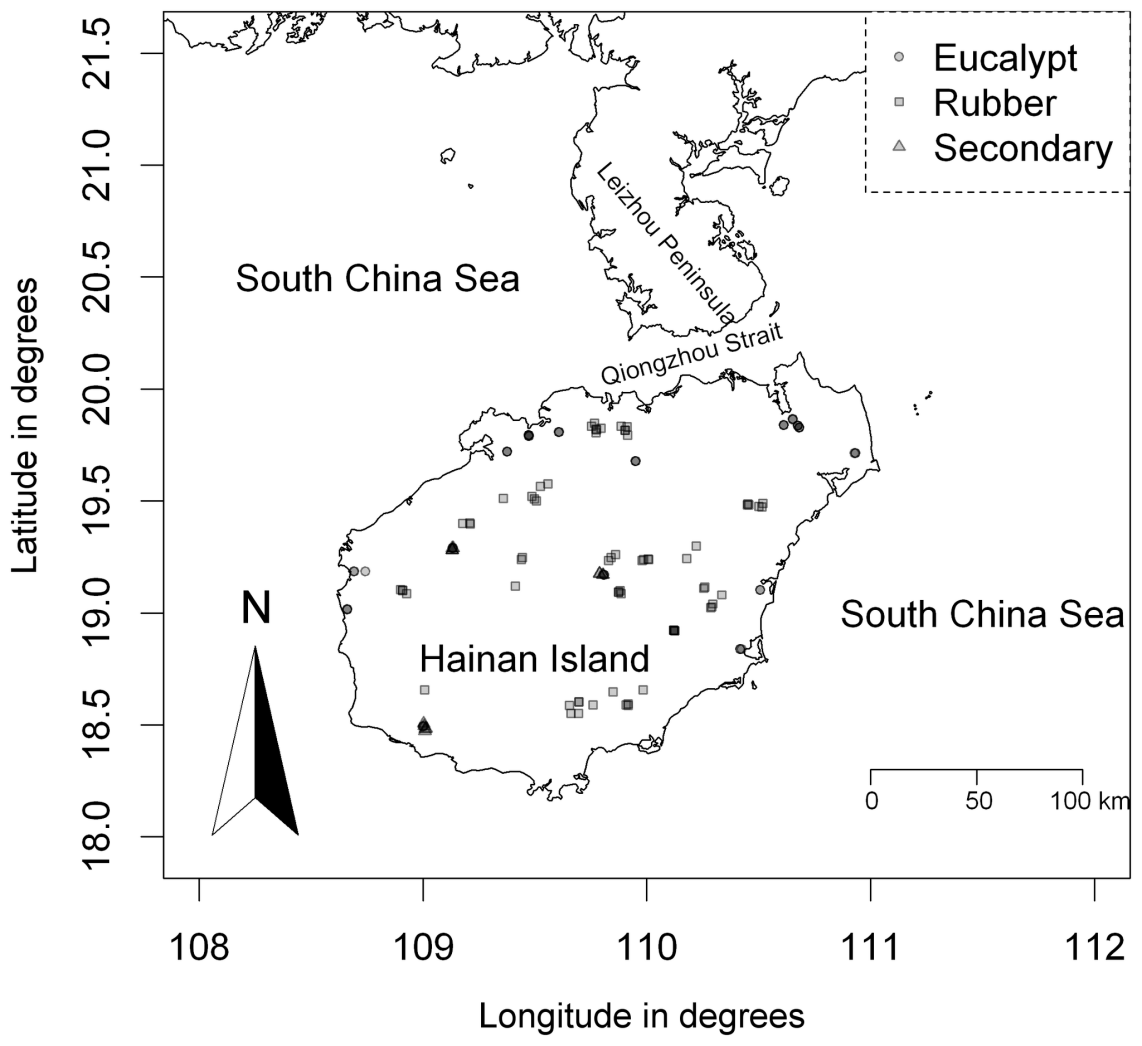
Distribution map of the 134 plots. Abbreviations: rubber (rubber plantation), eucalyptus (eucalyptus plantation), and secondary (secondary forest).

In the period from April to December in 2008, we established 134 plots in three types of tree communities, which included a rubber (*Hevea brasiliensis*) plantation (70 plots), a eucalyptus (*Eucalyptus urophylla*) plantation (50 plots), and a secondary forest (14 plots), in 10 counties spread throughout Hainan Island in southern China (Figure 1). We obtained the permission of the Hainan Jinhua Forestry Limited Company to establish plots in the two types of plantations and the permission of the Forestry Administration of Hainan Province to establish plots in the secondary forest. We did not record any endangered or protected species in the 134 plots. At each plot, a 30 × 30 m plot was established, and each tree standing with a diameter at breast height (DBH) of at least 1 cm was measured. The tree DBH data were used to compute the basal area of trees per square meter, which represented the biotic environmental variable for understory plant species. This variable was then used as an explanatory variable in the ordination models. We established five 2 × 2 m subplots located at the four corners and the center of a target 30 × 30 m plot. Within each subplot, all vascular and nonvascular plants with a height of at most 1.3 m were identified and counted to determine the number of individuals or clumps [26,39]. For a target 30 × 30 m plot, all of the understory plant species data of the five subplots (i.e. 2 × 2 m) were combined to represent its understory plant species composition. To evaluate the relative importance of each species, we computed an importance value for each species, defined as the sum of relative abundance and relative frequency.

To determine the effects of environmental variables, the altitude, annual rainfall, aspect, basal area of trees per square meter, canopy coverage, disturbance intensity, forest type, litter coverage, slope, soil moisture, slope position, soil texture, and thickness of litter were used as the environmental variables. Specifically, we used the Global Positioning System to measure the altitude of a target plot. The annual rainfalls of all of the plots were acquired from the records of local weather bureaus. The aspect of a target plot was estimated for one of the nine directions: the eight compass directions and flat. The basal area of the trees per square meter was calculated to represent the biomass reserves of tree species in a target plot. The canopy coverage and litter coverage of a target plot were estimated by field workers and recorded as percentages (ranging from 0% to 100%). Through interviews with local people, we recorded the disturbance intensity of a target plot. The disturbance intensities were grouped into four levels: weak, medium, strong, and super strong (coding as 1 to 4). The forest types were grouped in the following groups: rubber plantation, eucalyptus plantation, and secondary forest. We used a compass to measure the slope angles in degree at three random points and computed the average value as the slope. A topsoil (from a depth range of 0 to 10 cm) sample was collected from the center of each subplot, and we analyzed the soil moisture and texture; the average value of five soil moisture values of a target plot was defined as the soil moisture. The measurement of the soil moisture was conducted through gravimetry after the soil was dried at a maximum temperature of 105°C. Through particle size analysis of each soil sample, six soil texture classes were identified in the 134 plots: loam, silty loam, sandy loam, silt, and sand. We estimated the position of a plot relative to the mountain as the slope position and grouped these slope positions into four categories: bottom, low position, medium position, and top position (coding as 1 to 4). We randomly measured the thickness of the litter at three locations in a subplot; the average value of the 15 measurements of the thickness of the litter of a target plot was defined as the thickness of the litter for that plot.

### Partitioning variation in species richness and composition

To quantify the contribution of spatial and environmental heterogeneity on the variation in the species richness or composition of the understory plant species, the variation partitioning method was used [40]. According to the method described by Borcard, Gillet, and Legendre [41], variation partitioning based on multiple regression and redundancy analysis (RDA) were used to partition the variation in species richness and composition, respectively [42,43]. Before conducting the RDA on the species composition, the species composition data were transformed with the Hellinger transformation [44]. Specifically, the species composition data were constructed by counting the individuals of every understory species in every plot in the area of interest. Therefore, an n × p (plots-by-species) data table *X =* [*x*_*ij*_], where each *x*_*ij*_ element contains the number of individuals of understory species *j* in plot *i*, was formed. 

The 13 defined environmental variables were used to identify the effect of environmental heterogeneity on the species distributions. After the categorical variables were coded into dummy binary variables [41], 27 explanatory variables were generated. To model the spatial structures, principal coordinates of neighbor matrices eigenfunctions (PCNMs) were computed on the basis of a matrix of geographical distances [45]. 

The steps used to compute the PCNMs were the following. First, we converted the latitudes and longitudes of all the plots to Cartesian coordinates and then constructed a matrix of Euclidean distances for the 134 plots based on their new coordinates. Second, we used the maximum nearest neighbor distance as the threshold to truncate the matrix to retain only the distances between closely neighboring plots. All pairs of plots with distances that were higher than the threshold were replaced by four times the threshold. Third, we conducted a principal coordinate analysis of the truncated geographic distance matrix. Fourth, the PCNMs with positive eigenvalues were generated; specifically, 79 PCNMs were retained. The PCNMs, which represented the effect of space, were used as explanatory variables in the RDA.

In terms of the diagonalization of a spatial weighting matrix, the PCNM produced orthogonal maps that maximize the spatial auto-correlation; in fact, it creates PCNMs that can be directly linked to the spatial patterns of the environmental variables [46]. The PCNMs with positive eigenvalues were retained because these represented the positive spatial correlation. To avoid overfitting, forward selection was used to identify the significant environmental variables and PCNMs. First, we computed the globally adjusted R-square value based on all of the environmental variables or PCNMs. We then used this value as the threshold of the adjusted R-square value. Forward selection at the 5% significance level, while controlling for the adjusted R-square of the global model, was used to determine the most important explanatory variables that contribute to the variations in the species richness and composition [47].

### Multivariate regression tree (MRT) analysis

To identify the indicator species for the most important environmental variables, a MRT was conducted on the composition data of all species [48]. According to the MRT results on all species, indicator species of the groups defined by MRT analysis were identified and tested for significance through indicator species analysis [49]. 

### Diversity analysis among the three types of forests

By computing the alpha and beta diversity, we can identify how the plant species diversities of the three types of forests differ from each other. The directional species turnover and non-directional species variation are two main approaches that are used to define beta diversity [50]. In this study, there was no explicitly spatial, temporal, or environmental gradient among the study plots. Therefore, the variation in species composition is a rational way to quantify the beta diversity throughout the 134 plots [6]. Specifically, beta diversity is defined as the total variance in the Hellinger-transformed data table Y [6]:

BD=SS(Y)/(n−1) (1)

where *BD* is the beta diversity of the entire table and SS(Y) is the sum over all species and all plots of the squared deviations from the species means. To compare the beta diversity differences among the three types of forests, we randomly sampled 10 plots that do not overlap with each other from each type of forest. We then computed the beta diversity value of the 10 plots for each of the three types of forests and repeated this procedure 200 times. We then compared the beta diversity values of the three types of forests using the Kruskal-Wallis rank sum test. If a significant difference was found among the three groups, the pairwise Wilcoxon rank sum test was used to test the difference between each pair of groups.

To compare the alpha diversities among the three plots, the species richness was used to quantify the alpha diversity. Similarly, we randomly sampled 10 plots that do not overlap with each other from each type of forest. We then counted the species richness of the 10 plots for every type of forest and repeated this procedure 200 times. We compared the species richness among the three types of forests using the Kruskal-Wallis rank sum test. If a significant difference among the three groups was found, the pairwise Wilcoxon rank sum test was used to test the difference between each pair of groups.

### Exotic and native species

To identify the relationship between the exotic and the native species, we counted the richness of all exotic and native species in each of the 134 plots. We then analyzed the richness data for the exotic and native species through Kendall correlation tests for the 134 pairs of richness data. Similarly, we counted the number of individuals of all of the exotic and native species at each plot and then analyzed the abundance data of the exotic and the native species through Kendall correlation tests for the 134 pairs of abundance data. To separately identify the environment associations of exotic and native species, we independently conducted RDA on the species composition data of the exotic and the native species. After using the abovementioned random procedures to compare the differences in the beta diversity and richness, we then compared the differences in the beta diversity and richness between the exotic and the native species using the Kruskal-Wallis rank sum test. 

All of our analyses were conducted in the R (version 2.15.2) statistical language with labdsv, mvpart, MVPARTwrap, and packfor, PCNM, rdaTest and vegan packages [51].

## Results

### Community characteristics of the three types of vegetation

**Figure 2:**
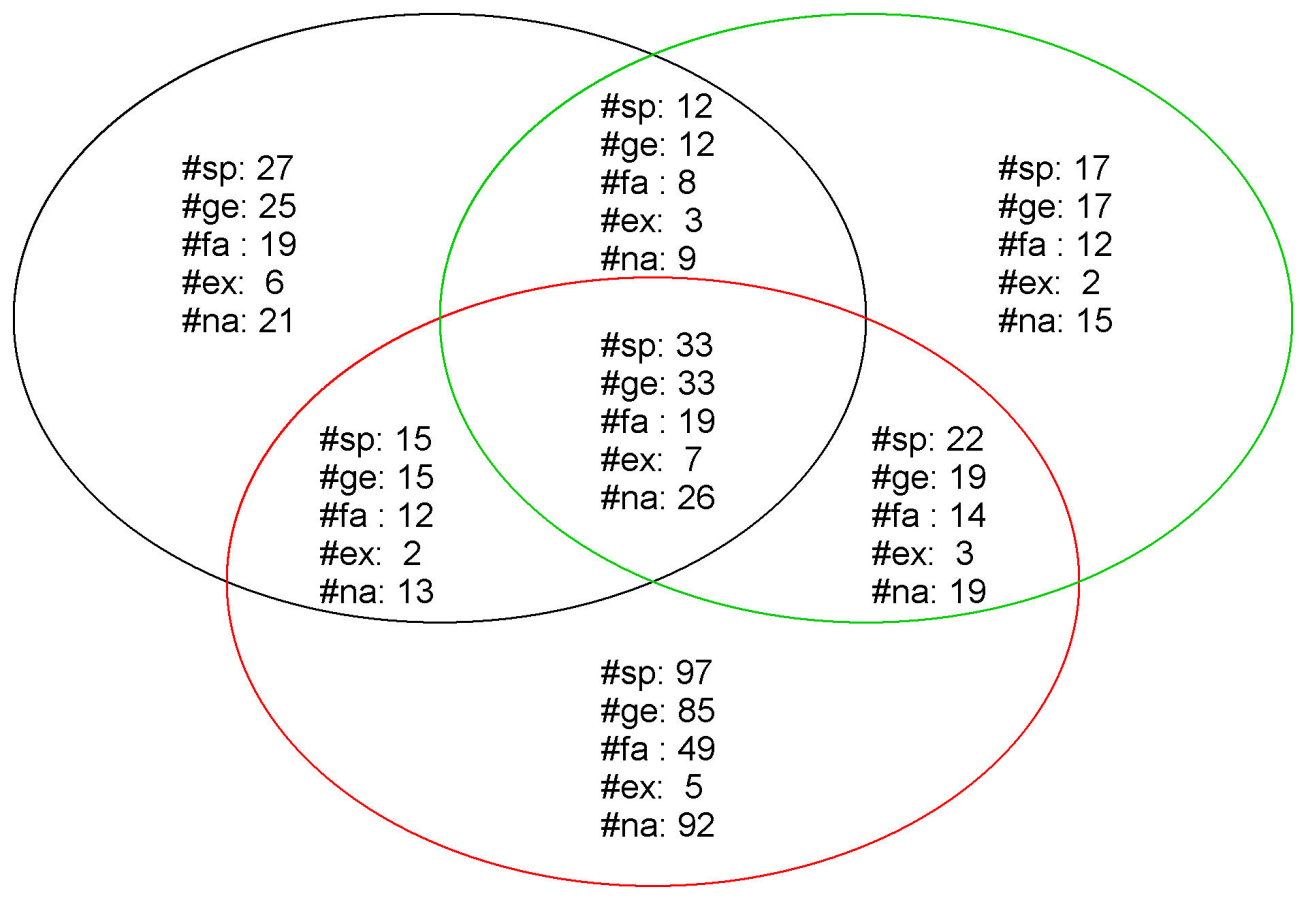
Venn diagram showing the species composition across the three types of forests. Black circle represents eucalyptus plantation, Green circle represents rubber plantation; and Red circle represents secondary forest. Abbreviations: #sp (number of species), #ge (number of genera), #fa (number of families), #ex (numer of exotic species), and #na (number of native species).

In the understory of the 134 plots, we identified 223 morphospecies, which belonged to 180 genera and 75 families. Specifically, 84, 87, and 167 species were recorded in the rubber plantation, the eucalyptus plantation, and the secondary forest, respectively (Figure 2). As shown in Table 1, the community composition of the rubber plantation is clearly different from that of the eucalyptus plantation. The most abundant species in the rubber plantation was *Acroceras munroanum*, which is a native species of Hainan Island. In contrast, the most abundant species in the eucalyptus plantation was *Praxelis clematidea*, which is an exotic species. In the 134 plots, the 12 species that exhibited an abundance greater than 1,000 were the following (written in descending order): *A. munroanum, P. clematidea, Borreria stricta, Imperata cylindrical, Arthraxon prionodes, Axonopus compressus, Dactyloctenium aegyptium, Tridax procumbens, Dicranopteris linearis, Ichnanthus vicinus, Neottopteris antrophyoides*, and *Chromolaena odorata*.

**Table 1:** Top 10 understory species of importance value rank for each of the three forest types.

Vegetation type	Species	Importance value	Species type
Rubber plantation	*Acroceras munroanum*	28.26	Native
	*Borreria stricta*	7.67	Native
	*Axonopus compressus*	5	Exotic
	*Ichnanthus vicinus*	4.2	Native
	*Chromolaena odorata*	3.25	Exotic
	*Pteris cretica* var.nervosa	3.06	Native
	*Dicranopteris linearis*	2.9	Native
	*Neottopteris ntrophyoides*	2.83	Native
	*Lygodium japonicum*	2.5	Native
	*Praxelis clematidea*	2.29	Exotic
Eucalypt plantation	*Eupatorium catarium*	19.06	Exotic
	*Imperata cylindrical*	5.17	Exotic
	*Arthraxon prionodes*	4.94	Native
	*Chromolaena odorata*	3.42	Exotic
	*Borreria stricta*	1.8	Native
	*Acroceras munroanum*	1.73	Native
	*Rhodomyrtus tomentosa*	1.4	Native
	*Melastoma malabathricum*	1.35	Exotic
	*Urena lobata* Linn.	1.17	Exotic
	*L. japonicum*	1.15	Native
Secondary forest	*E. catarium*	6.45	Exotic
	*Borreria stricta*	3.66	Native
	*Tridax procumbens*	2.36	Exotic
	*Dactyloctenium aegyptium*	2.28	Native
	*Acroceras munroanum*	1.08	Native
	*R. tomentosa*	0.59	Native
	*Digitaria sanguinalis*	0.56	Native
	*Miscanthus floridulus*	0.53	Native
	*Psychotria rubra*	0.47	Native
	*Eurya nitida*	0.42	Native

### Variation partitioning results

**Figure 3:**
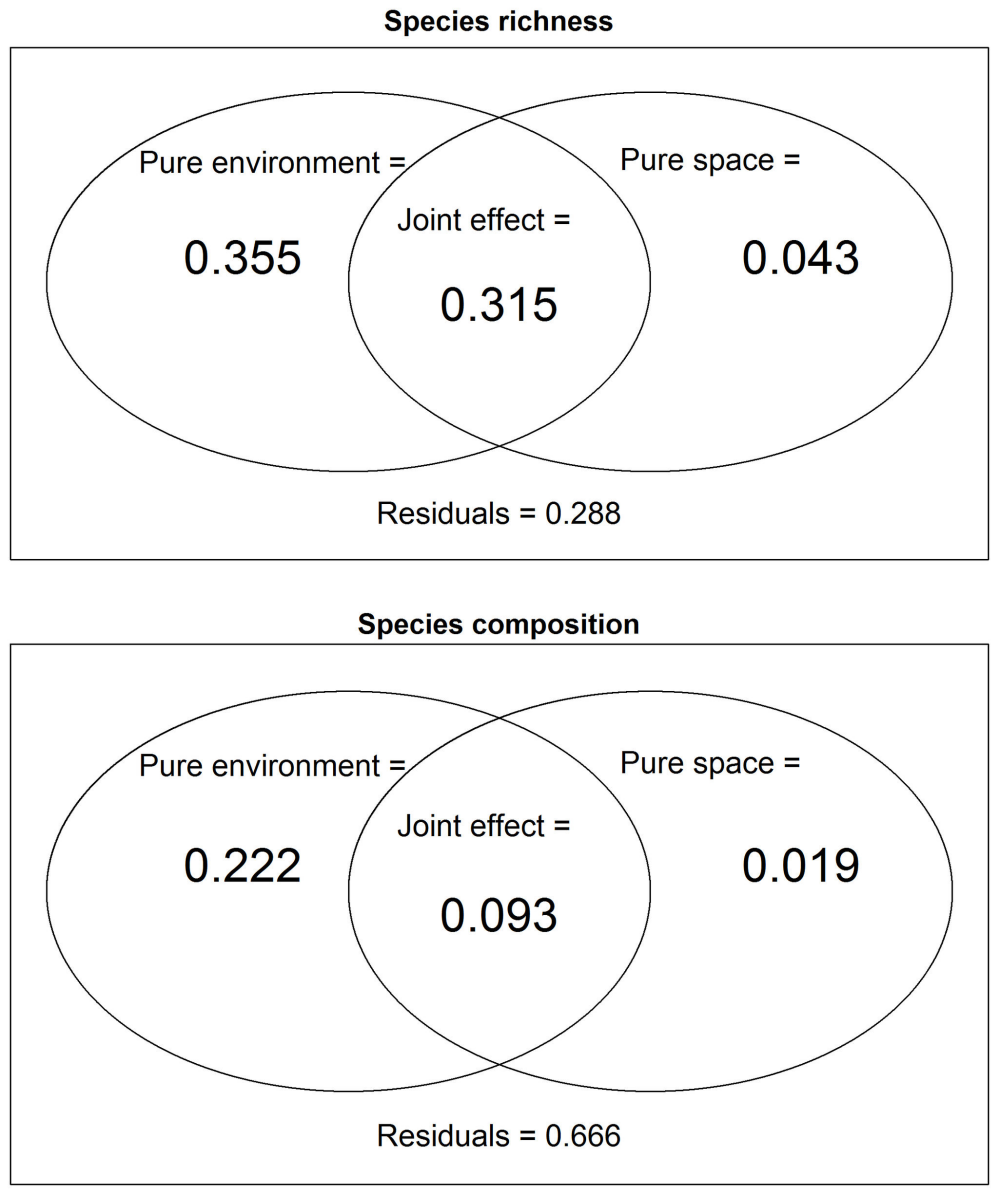
Variation partitioning results of all species. The two figure panels show Venn diagrams that represent the partitioning of the variations of the species richness and composition constrained by the selected environmental variables (environment) and PCNMs (space). Each box represents 100% of the variation in the corresponding response variable. The fractions shown are the adjusted R-square statistics.

**Figure 4:**
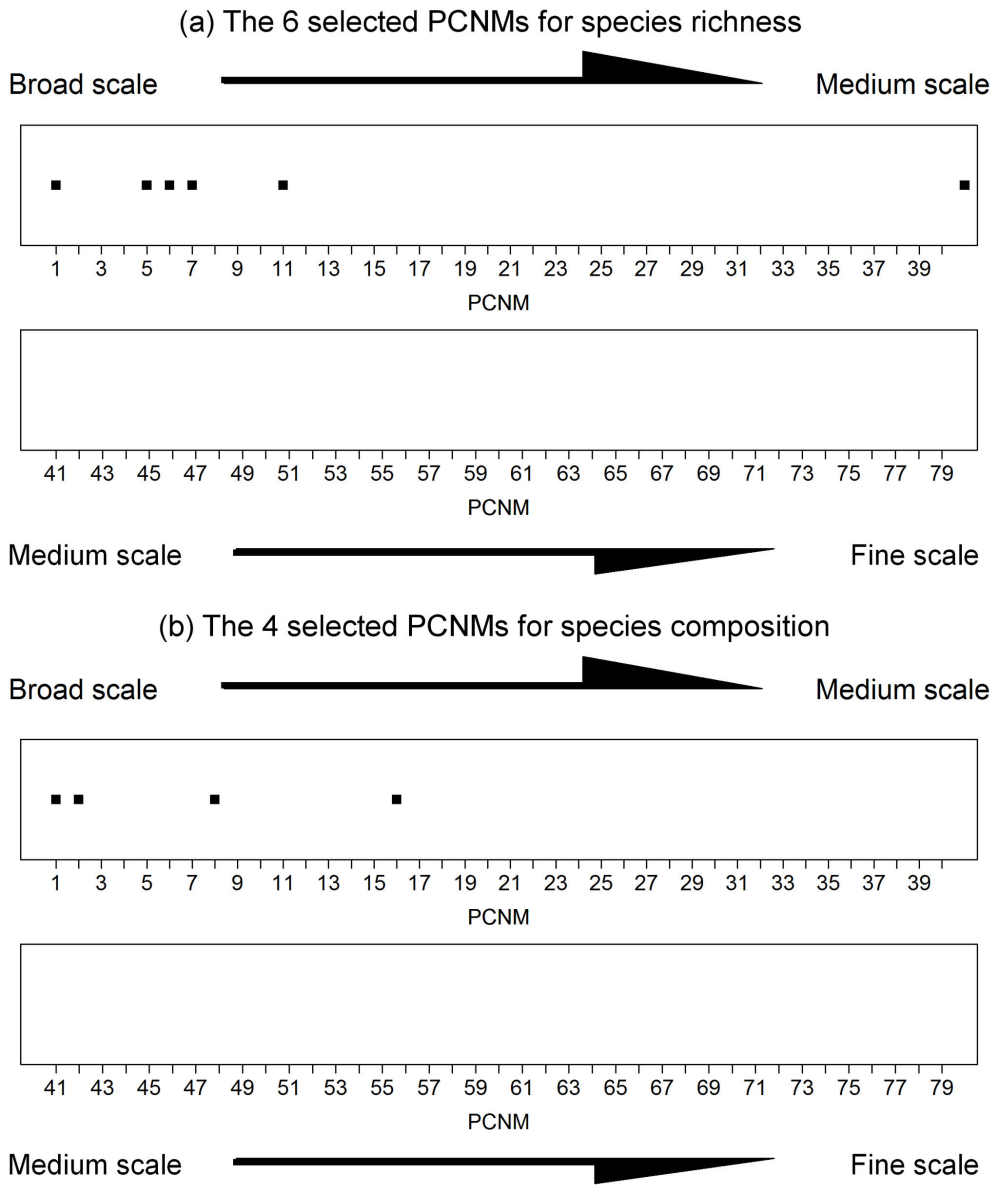
Selected PCNMs from the 79 PCNMs that exhibit a positive spatial correlation for the richness and composition of all species. The PCNMs are represented by square dots.

The variation partitioning results showed that the environmental heterogeneity explained a larger proportion of the variations in the species richness and the species composition (Figure 3). In particular, six environmental variables were selected through forward selection of the RDA of the species richness constrained by the environmental variables. The selected factors (in descending order of relative importance) were the following: secondary forest, altitude, slope position, disturbance intensity, silty loam, and soil moisture. Even the RDA of the species richness constrained by both environmental variables and the PCNMs revealed that secondary forest still plays the most important role in the determination of the species richness pattern. As shown in Figure 4, the PCNMs selected to explain spatial structure of species richness and composition are mostly broad-scale ones.

**Figure 5:**
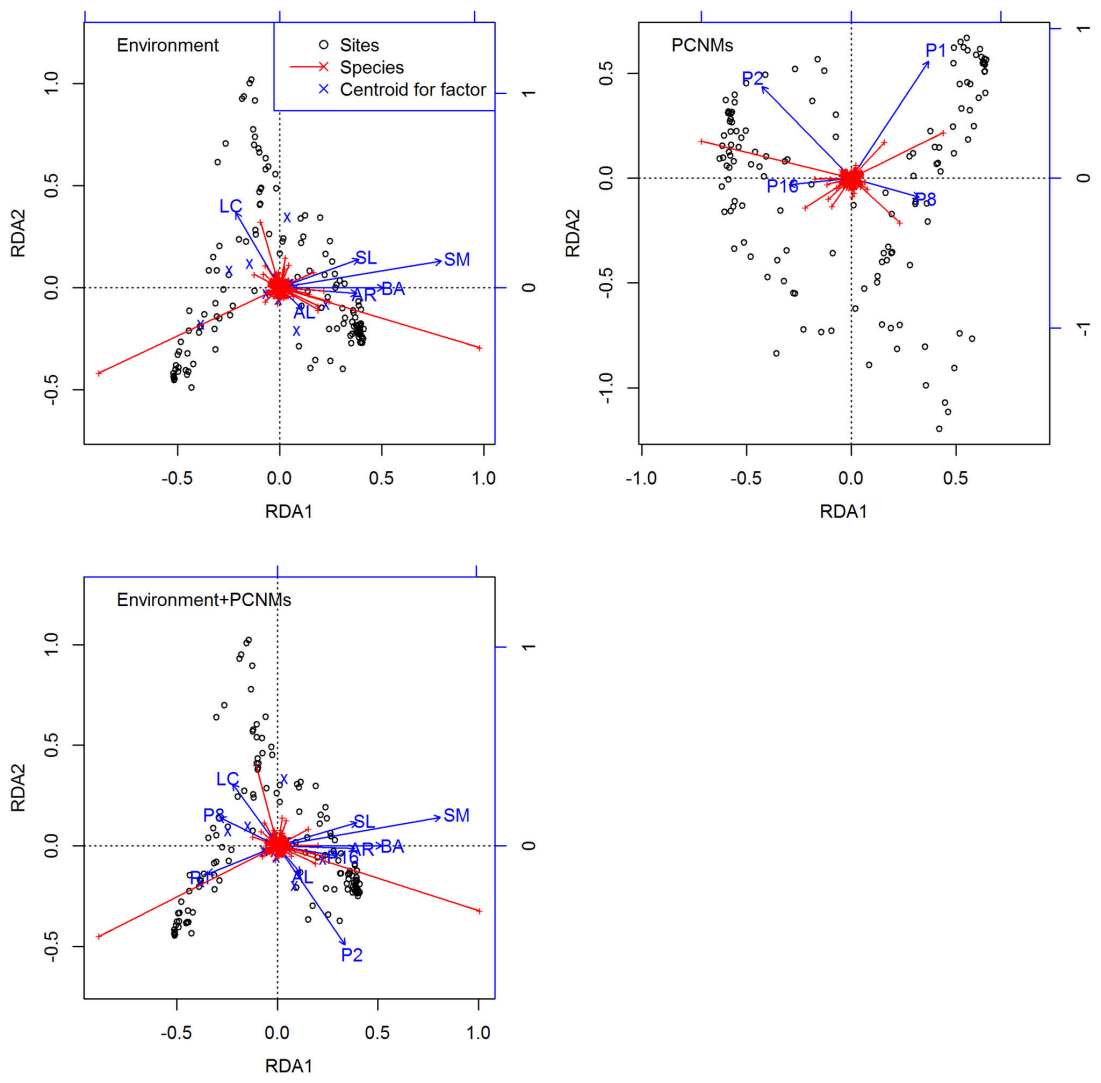
RDA tri-plots of the species composition data constrained by the selected environmental variables and PCNMs, scaling 2. Abbreviations: AL (altitude), AR (annual rainfall), BA (basal area of trees per square meter), LC (litter coverage), SL (slope), SM (soil moisture). The bottom and left-hand scales are for the objects and the response variables, the top and right-hand scales are for the explanatory variables.

When the species composition was constrained only by the environmental variables, the RDA results demonstrated that the rubber plantation plays the most important role in the determination of the pattern of the species composition. The six most important environmental factors that determine the species composition (in descending order) were the following: rubber plantation, soil moisture, sand soil texture, basal area of trees, sandy loam soil texture, and litter coverage. Figure 5 shows the associations among environmental variables, plots and species. When the species composition was constrained by the pure space of the PCNMs, PCNM1, which belongs to the broad scale, exhibited the strongest effect on the species composition (Figure 5). The rubber plantation and soil moisture still played the most and the second most dominant roles in the determination of the species distributions when the species composition was constrained by the joint effect of the environmental variables and the PCNMs. 

### MRT results

**Figure 6:**
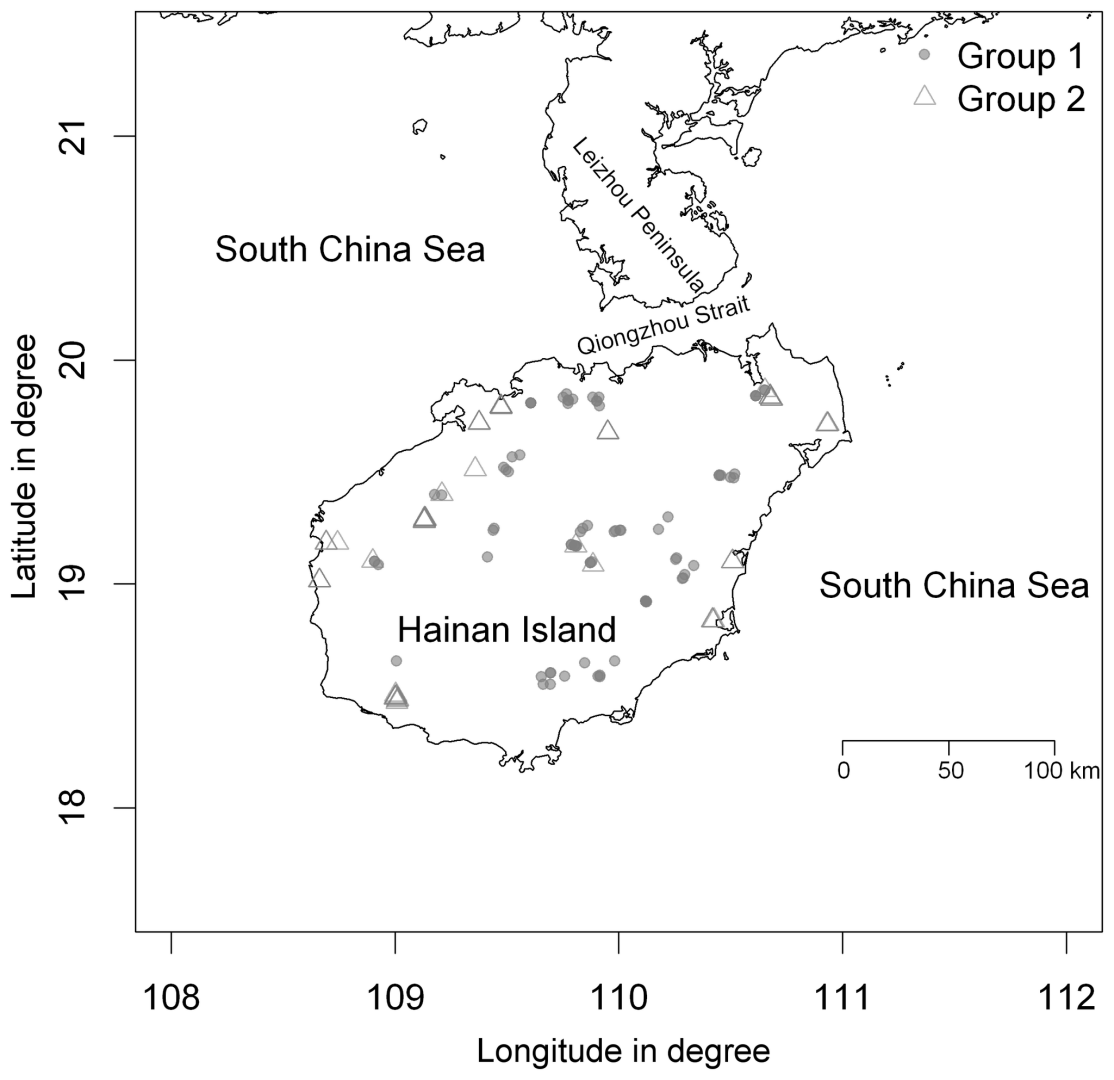
Clustering with the soil moisture constraint using MRT. Group 1 represents the sites with soil moisture greater than 11.55%. Group 2 represents those sites with soil moisture less than 11.55%.

For all species, the best solution with the smallest cross-validated relative error (CVRE) of the MRT analysis generated two groups for the 134 plots (Figure 6). Of all the environmental variables, the soil moisture was identified as the most important factor that determines the species distributions. Of the 223 species (Table S1), *Eupatorium catarium* and *Acroceras munroanum* were identified as the most indicative species of the two MRT groups. Specifically, *A. munroanum* and *E. catarium* represent the two groups of species that prefer wet and dry habitats, respectively. Other than 2 groups, Figure S1 showed that solutions with 3 to 9 groups also presented good performance during the cross-validation procedure. The R-square values of the solutions from 2 to 9 in ascending order were 18.9%, 23.4%, 26.4%, 29.4%, 32.3%, 34.8%, 37.0%, 39.0%.

**Figure 7:**
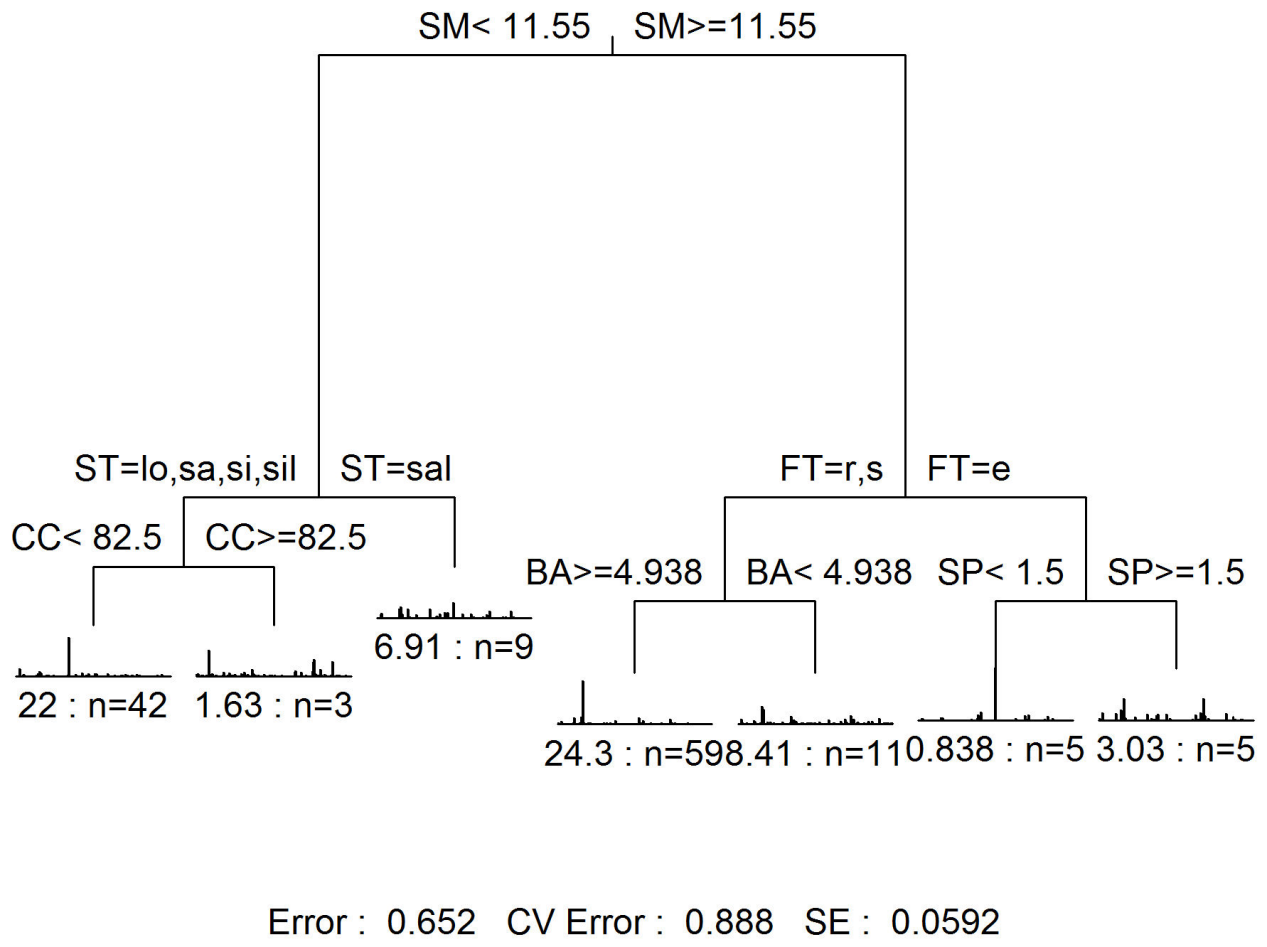
The solution of MRT tree with 7 groups for all species. Abbreviations: SM (soil moisture), CC (canopy coverage), ST=lo,sa,si,sil (soil texture in loam, sand, silt and silty loam), ST=sal (soil texture in sandy loam), BA (basal area of trees per square meter), FT=r,s (forest type in rubber plantation and secondary forest), FT=e (forest type in eucalyptus plantation), SP (slope position), # : n (relative error : number of sites), CV (cross-validation), SE (standard error).

The solution with 7 groups presented the second best performance among all the solutions (Figure S1). In most cases of this solution, the more sites in each group, the larger relative error would be (Figure 7). Table 2 shows the species composition summary in each of the 7 groups. The MRT tree nodes in Figure 7 present how environmental variables characterized the understory species and plots composition in each group. For instance, the third group (MRT tree leaves ranking from left to right in Figure 7) was formed when soil moisture was less than 11.55% and soil texture equaled sandy loam. After conducting an indicator analysis on this MRT solution, we identified 1, 54, 3, 1, 2, 4 and 9 indicator species (with P-value less than 0.05) for the groups from 1 to 7 sequentially. Specifically, the indicator species with the largest indicator species value from group 1 to 7 were *Eupatorium catarium*, *Fissistigma glaucescens*, *Aristida adscensionis*, *Acroceras munroanum, Cratoxylum cochinchinense*, *Arthraxon prionodes* and *Breynia fruticosa*, sequentially.

**Table 2:** The species composition in each of the 7 groups of the MRT solution on all species.

Group	Number of species	Number of families	Number of genera	Number of exotic species	Number of native species	Number of sites
1	113	48	99	25	88	42
2	68	40	62	0	68	3
3	35	20	35	6	29	9
4	81	37	73	15	66	59
5	80	39	67	10	70	11
6	17	12	17	6	11	5
7	37	20	36	6	31	5

Note: The group numbers are corresponding to the 7 groups in Figure 7 from left to right sequentially.

### Diversity differences among the three types of forest

**Figure 8:**
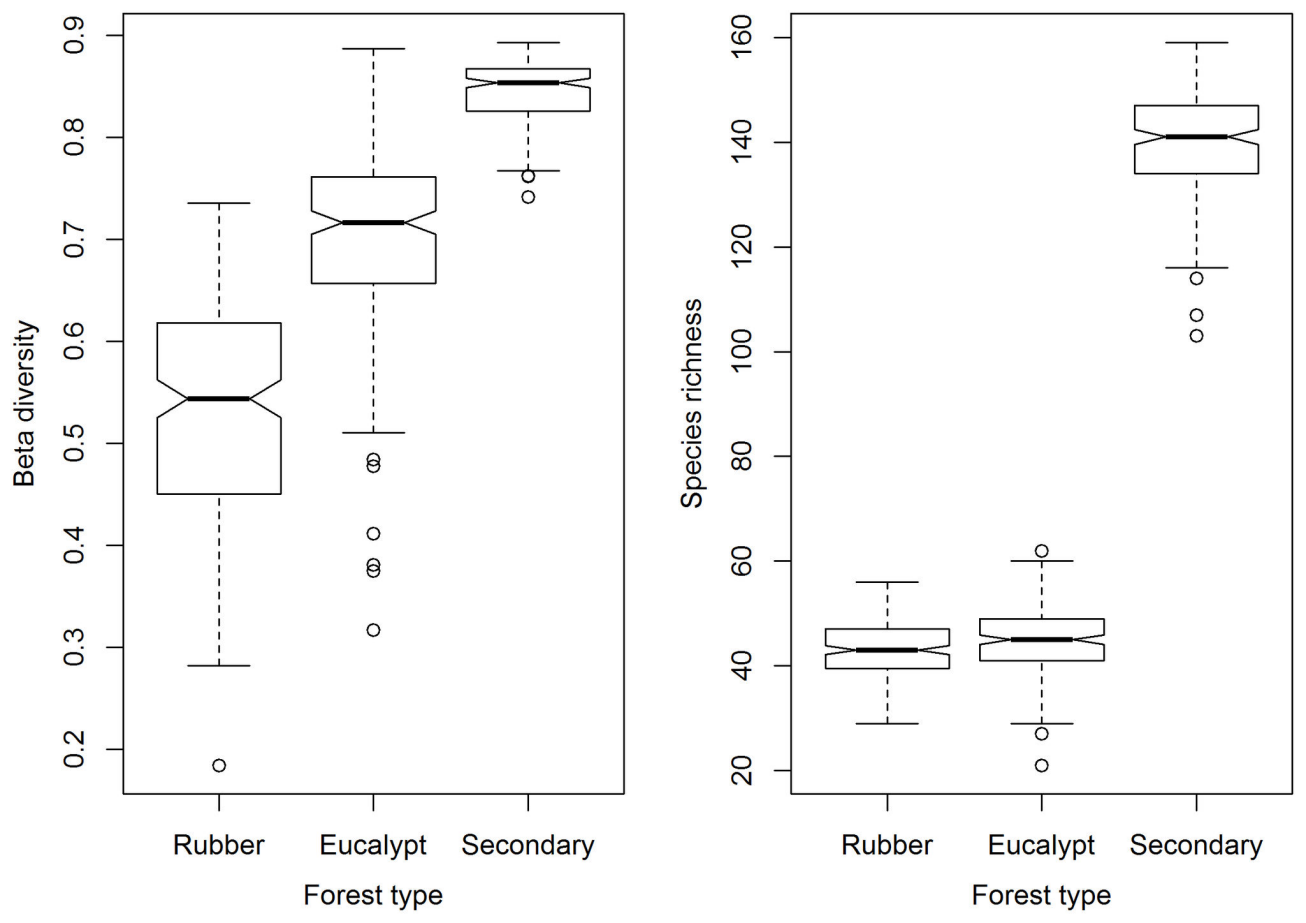
Boxplots of the alpha and beta diversity values for the three types of forests.

The P-value of the Kruskal-Wallis rank sum test on the beta diversities among the three types of forests was less than 2.2e-16. Thus, we further conducted pairwise Wilcoxon rank sum test on beta diversities of the three pair of forest types, the P-values of the three pairs of forest types were all less than 2e-16. On the other hand, the P-value of the Kruskal-Wallis rank sum test on the alpha diversities among the three types of forests was also less than 2.2e-16. Thus, we further conducted pairwise Wilcoxon rank sum test on alpha diversities of the three pair of forest types, the P-values of the between the two plantation was 0.00016, the P-values of other two pairs of forest types were both less than 2e-16. Therefore, there were significant alpha and beta diversity differences among all the three forest types. As shown in Figure 8, both the alpha (richness) and the beta diversities of understory plant species of secondary forest were significantly higher than that of the other two types of forests. Moreover, the beta diversities of the three types of forests exhibited a significantly decreasing tendency in the following order: secondary forest, eucalyptus plantation, and rubber plantation.

### Exotic and native species

The results of the Kendall correlation tests between the richness data of exotic and native species revealed no significant correlation between the two types of species. In addition, there was also no significant correlation between the abundance data of the two types of species. However, when we removed the 3 plot data marked with blue circles (Figure S2A), and conducted Kendall correlation test between the richness data of exotic and native species again, Kendall's tau was -0.356 (with *P*-value 1.961e-09). This indicated a remarkable negative association between the abundance data of exotic and native species (Figure S2B). We further conducted Wilcoxon rank sum and signed rank tests on each environmental variables between the three outlier plots and the remaining plots and found that the three plots were all secondary vegetation with thin thickness of litter significantly different from other plots (with P-value 0.0175 and 0.0026 for thickness of litter and forest type, respectively). The P-value of the Kruskal-Wallis rank sum test on the beta diversity between exotic and native species community was less than 2.2e-16. Similarly, the P-value of the Kruskal-Wallis rank sum test on the alpha diversity between exotic and native species community was less than 2.2e-16 too. As shown in Figure S3, the alpha and beta diversities of native species were significantly higher than those of exotic species. 

After conducting an RDA on the richness data of exotic species constrained by the environmental factors, five factors were selected through forward selection (shown in descending order of relative importance): canopy coverage, northeast aspect, altitude, loam, and slope. The five selected factors constrained 37.9% of the variations in the richness data of exotic species. Using the richness data of native species, six factors were selected by RDA (in descending order of relative importance): secondary forest, slope, canopy coverage, northeast aspect, loam, and soil moisture. These six selected factors constrained 70% of the variations in the richness data of native species. The environmental variables always constrained a larger proportion of the richness data than the PCNMs (Figure S4).

An RDA has been separately computed on the exotic and native species compositions constrained by the environmental factors. Figure S5 shows the associations among environmental variables, plots and species in the communities of exotic and native species. The six most important factors that determine the composition patter of exotic species (in descending order) were the soil moisture, rubber plantation, basal area of trees per square meter, annual rainfall, slope, and sandy loam soil texture. The six most important factors that determine the composition of native species (in descending order) were the following: rubber plantation, soil moisture, litter coverage, basal area of trees per square meter, sand soil texture, and slope. Through separate variation partitioning with RDA on the composition data of exotic and native species between the environmental variables and the PCNMs, we found that the environmental variables always outperform the PCNMs (Figure S6).

## Discussion

Numerous studies have studied the associations of species spatial patterns with the space and environment at the local [7], meso [10], and landscape scales [52]. Using understory plant species data across Hainan Island, we found that the environment (biotic and abiotic factors) plays a major role in the determination of both the richness and composition of understory plant species at the landscape scale. Of the biotic and abiotic variables studied, the overstory composition usually plays the leading role in the determination of the species richness and composition patterns. The different habitat associations of common and rare species explain the species assembly mechanism differences to some extent. The different diversity patterns among the three types of forests and between the two types of species suggest that it is critical to formulate a proper strategy to ensure the maintenance of species diversity.

### Species richness

In this study, the environmental variables predominantly explained the richness pattern of all understory plant species, especially for native species (Figures 3 and S4). This result is different from the previous finding that PCNMs predominantly constrain the richness patterns of tree species at the local scale (

< 1 km^2^) [7]. By summarizing previous empirical studies [27–29,52,53], John et al. [19] argued that environmental factors are likely to play a deterministic role in the regulation of species diversity at the large meso and landscape scales. This provides strong evidence that the different scales used explain why our result is markedly different from the results reported by Legendre et al. [7]. Of all the biotic and abiotic factors studied, the secondary forest was found to play the most important role in the shaping of the richness pattern of all species. Although the number of secondary forest plots only accounts for a small part (11.2%) of the total number of plots, the number of species in the secondary forest plots accounts for 74.9% of the total number of species. Thus, it is clear why the secondary forest variable explains a large amount of the variations in the species richness data. A previous study reported that the overstory composition dramatically influences the understory plant species richness and composition [22], and we also found that the overstory composition does exert strong effects on the species diversity pattern. To summarize, we conclude that environmental factors, particularly the overstory composition (secondary forest), play the principal role

 in the shaping of the alpha diversity of understory plant species at the landscape scale.

### Species composition

Similar to the variation partitioning results obtained from the analysis of species richness patterns, the environmental variables outperform the space variables in the analysis of the species composition data. In the analysis of all understory plant species, the analysis of native species, and the analysis of exotic species, the variation partitioning results always exhibit the same patterns (Figures 3 and S6). This finding suggests that niche differentiation is the major force that drives the beta diversity pattern of the understory plant species in Hainan Island. Our results are consistent to those obtained in studies that disentangle the effect of space, which is modeled using the geographic distance, and the environment on the boreal understory plant species and the Amazonian forest plant species at the mesoscale and the landscape scale [8,52]. However, these results are different from those obtained in studies that used PCNMs to explore the effect of space and environment on pteridophyte and tree species composition data at both the mesoscale and the local scale [7,9]. Similarly to the variation partitioning of the species richness patterns, the overstory composition plays the most important role in the determination of the spatial patterns of the understory plant species composition. With the exception of the RDA results obtained for exotic species, the most dominant factors were always found to be the rubber plantation. 

### Relative importance of environmental variables

The RDA results of the understory plant species richness and composition are basically in agreement with previous findings that showed the importance of the overstory composition, site condition (e.g., soil texture and slope), and climate on the understory plant species composition spatial patterns [22]. Specifically, we found that the rubber plantation and secondary forest predominantly determine the understory plant species composition and richness patterns, respectively. Through modification of resources, such as light and soil nutrient availability [25,54,55], the overstory trees determine the composition of the understory plant species. With respect to the site conditions, Chen et al. [26] discovered that the soil moisture is critical for the understory composition in temperate forests, and we further show that this is also true for tropical forests (Table S1). Moreover, the RDA results of all understory plant species, of the native species, and of the exotic species confirm that the physical site conditions, such as soil texture and slope, are of secondary importance in Hainan Island (Figures 5 and S5). In addition, the litter coverage and the basal area of trees per square meter, which reflect the competition of nutrients, were always included by the models. However, Chen et al. [26] found that the contribution of the tree basal area to the understory plant species composition is limited in temperate old-growth forests. To obtain a general idea of how the tree basal area influences the understory plant species compositions in tropical and temperate forests, a future supplementary study should identify the tree basal area effect on the distribution patterns of understory plant species in a tropical primary forest. The analysis of the climate-related variables revealed that the annual rainfall contributes to the distribution patterns of exotic understory plant species but not to that of native species. Taken together, the data show that the environmental variables that were introduced into the models determine the understory plant species distributions and that these are essential niche axes for the coexistence of understory plant species.

### Diversity patterns

It is critical to protect secondary forests in order to maintain the species diversity of native understory plant species. The alpha and beta diversities of understory plant species of secondary forests are significantly higher than that of the plantations. Because secondary forests, of the three types of forests studied, may exhibit the most similar species composition of native understory plants compared with primary forests, the protection of the secondary forest may aid the recovery of the primary forest. Moreover, 90% (150) of the understory plant species are native species of the secondary forest and account for 76.9% of all of the native species recorded in the 134 plots across Hainan Island. As a result, the protection of the secondary forest is beneficial for the recovery and maintenance of the native plant species diversity. However, the understory of secondary forests is dominated by exotic species (Table 1). Due to the significantly negative association between the abundance of native species and the abundance of exotic species (Figure S2), the ecosystem functions of native species is remarkably limited by exotic species. To recover the ecosystem functions of native species in the secondary forest, it is necessary to undertake proper management strategies to decrease the abundance of exotic species or even completely remove these. The MRT results show that the most abundant exotic species (*Eupatorium catarium*) prefers a dry habitat (soil moisture < 11.55%). Of the 14 secondary forest plots studied, the soil moisture of 10 of the plots is less than 11.55%. In the four wet secondary plots, the abundances of exotic and native species were found to be 40 and 1,085, respectively; the abundances of all exotic and native understory species in the 10 dry plots of secondary forest were found to be 6,280 and 5,866, respectively. To summarize, the identification of a management strategy to increase the soil moisture of secondary forests might help protect most of the native understory plant species in Hainan Island forests.

## Conclusions

The environmental heterogeneity dominantly structures the distribution patterns of the richness and composition of understory plant species at the landscape scale. Of all the environmental factors and PCNMs analyzed, the overstory composition (forest type) was found to always play the leading role in the determination of the richness and composition patterns of the understory plants. Among the spatial structures, broad scale ones outperform medium and fine scale ones in shaping species richness and composition patterns. The MRT analysis of the species diversity revealed that the soil moisture might be the key to the maintenance of most of the native species diversity. The alpha and beta diversities of the secondary forest plots were remarkably higher than that of the two plantations. We conclude that niche assembly is the key mechanism regulating understory plant species distributions in Hainan Island.

## Supporting Information

Figure S1 **Graph of the relative error and the CVRE for MRT results of all species.** The solution with the smallest CVRE is indicated (yellow point), as well as CVRE error bars. The green bars indicate the number of times that the solution was selected as the best during the cross-validation iterations.
(TIF)

Figure S2 **The relationship between the abundances of exotic and native species before and after removing the 3-plot data.** The filled blue circles represent the plots that were removed.
(TIF)

Figure S3 **Boxplots of the differences in the beta and alpha diversity values between exotic and native species.**
(TIF)

Figure S4 **Variation partitioning results of the exotic and native species richness.** The two figure panels show Venn diagrams that represent the partitioning of the variations of the richness of the exotic and the native species constrained by the selected environmental variables (environment) and PCNMs (space). The conventions are the same as in Figure 3.
(TIF)

Figure S5 **RDA tri-plots of the exotic and native species composition data separately constrained by the selected environmental variables, scaling 2.** Abbreviations: AL (altitude), AR (annual rainfall), BA (basal area of trees per square meter), LC (litter coverage), SL (slope), SM (soil moisture), SP (slope position). The bottom and left-hand scales are for the objects and the response variables, the top and right-hand scales are for the explanatory variables.
(TIF)

Figure S6 **Variation partitioning results of the exotic and native species composition.** The two figure panels show Venn diagrams that represent the partitioning of the variations of the exotic and the native species compositions constrained by the selected environmental variables (environment) and PCNMs (space). The conventions are the same as in Figure 3.
(TIF)

Tables S1 **Indicator species for the two MRT groups of all understory plant species.**
(DOC)

## References

[1] Condit R, Pitman N, Leigh EG, Chave J, Terborgh J et al. (2002) Beta-diversity in tropical forest trees. Science 295: 666–669. doi:10.1126/science.1066854. PubMed: 11809969. 11809969

[2] Gotelli NJ, Anderson MJ, Arita HT, Chao A, Colwell RK et al. (2009) Patterns and causes of species richness: a general simulation model for macroecology. Ecol Lett 12: 873–886. doi:10.1111/j.1461-0248.2009.01353.x. PubMed: 19702748. 19702748

[3] Wright SJ (2002) Plant diversity in tropical forests: a review of mechanisms of species coexistence. Oecologia 130: 1–14. 28547014 10.1007/s004420100809

[4] Legendre P, Borcard D, Peres-Neto PR (2005) Analyzing beta diversity: partitioning the spatial variation of community composition data. Ecol Monogr 75: 435–450. doi:10.1890/05-0549.

[5] Hubbell SP (2001) The unified neutral theory of biodiversity and biogeography. Princeton, New Jersey, USA: Princeton University Press. 448pp.

[6] De Cáceres M, Legendre P, Valencia R, Cao M, Chang LW et al. (2012) The variation of tree beta diversity across a global network of forest plots. Global Ecol Biogeogr 21: 1191–1202. doi:10.1111/j.1466-8238.2012.00770.x.

[7] Legendre P, Mi X, Ren H, Ma K, Yu M et al. (2009) Partitioning beta diversity in a subtropical broad-leaved forest of China. Ecology 90: 663–674. doi:10.1890/07-1880.1. PubMed: 19341137. 19341137

[8] Gilbert B, Lechowicz MJ (2004) Neutrality, niches, and dispersal in a temperate forest understory. Proc Natl Acad Sci U S A 101: 7651–7656. doi:10.1073/pnas.0400814101. PubMed: 15128948. 15128948 PMC419661

[9] Jones MM, Tuomisto H, Borcard D, Legendre P, Clark DB et al. (2008) Explaining variation in tropical plant community composition: influence of environmental and spatial data quality. Oecologia 155: 593–604. doi:10.1007/s00442-007-0923-8. PubMed: 18064493. 18064493

[10] Jones MM, Tuomisto H, Clark DB, Olivas P (2006) Effects of mesoscale environmental heterogeneity and dispersal limitation on floristic variation in rain forest ferns. J Ecol 94: 181–195. doi:10.1111/j.1365-2745.2005.01071.x.

[11] Lekberg Y, Koide RT, Rohr JR, Aldrich-Wolfe L, Morton JB (2007) Role of niche restrictions and dispersal in the composition of arbuscular mycorrhizal fungal communities. J Ecol 95: 95–105. doi:10.1111/j.1365-2745.2006.01193.x.

[12] Novotny V, Miller SE, Hulcr J, Drew RAI, Basset Y et al. (2007) Low beta diversity of herbivorous insects in tropical forests. Nature 448: 692–695. doi:10.1038/nature06021. PubMed: 17687324. 17687324

[13] Muneepeerakul R, Bertuzzo E, Lynch HJ, Fagan WF, Rinaldo A et al. (2008) Neutral metacommunity models predict fish diversity patterns in Mississippi-Missouri basin. Nature 453: 220–222. doi:10.1038/nature06813. PubMed: 18464742. 18464742

[14] Gilliam FS, Turrill NL (1993) Herbaceous layer cover and biomass in a young versus a mature stand of a central Appalachian hardwood forest 120. Bull Torrey Bot Club pp. 445–450. PubMed: 8279789.

[15] Halpern CB, Spies TA (1995) Plant species diversity in natural and managed forests of the Pacific Northwest. Ecol Appl 5: 913–934. doi:10.2307/2269343.

[16] Hu YH, Sha LQ, Blanchet FG, Zhang JL, Tang Y et al. (2012) Dominant species and dispersal limitation regulate tree species distributions in a 20-ha plot in Xishuangbanna, southwest China. Oikos 121: 952–960. doi:10.1111/j.1600-0706.2011.19831.x.

[17] Shen G, Yu M, Hu XS, Mi X, Ren H et al. (2009) Species-area relationships explained by the joint effects of dispersal limitation and habitat heterogeneity. Ecology 90: 3033–3041. doi:10.1890/08-1646.1. PubMed: 19967859. 19967859

[18] Wang X, Wiegand T, Wolf A, Howe R, Davies SJ et al. (2011) Spatial patterns of tree species richness in two temperate forests. J Ecol 99: 1382–1393. doi:10.1111/j.1365-2745.2011.01857.x.

[19] John R, Dalling JW, Harms KE, Yavitt JB, Stallard RF et al. (2007) Soil nutrients influence spatial distributions of tropical tree species. Proc Natl Acad Sci U S A 104: 864–869. doi:10.1073/pnas.0604666104. PubMed: 17215353. 17215353 PMC1783405

[20] Barbier S, Gosselin F, Balandier P (2008) Influence of tree species on understory vegetation diversity and mechanisms involved-A critical review for temperate and boreal forests. Forest Ecol Manage 254: 1–15. doi:10.1016/j.foreco.2007.09.038.

[21] Comita LS, Uriarte M, Thompson J, Jonckheere I, Canham CD et al. (2009) Abiotic and biotic drivers of seedling survival in a hurricane-impacted tropical forest. J Ecol 97: 1346–1359. doi:10.1111/j.1365-2745.2009.01551.x.

[22] Hart SA, Chen HYH (2006) Understory vegetation dynamics of North American boreal forests. Crc. Crit Rev Plant Sci 25: 381–397. doi:10.1080/07352680600819286.

[23] Queenborough SA, Burslem DF, Garwood NC, Valencia R (2009) Taxonomic scale-dependence of habitat niche partitioning and biotic neighbourhood on survival of tropical tree seedlings. Proc Royal Soc of London B-Biol Sci 276: 4197–4205. doi:10.1098/rspb.2009.0921. PubMed: 19740886. PMC2821336 19740886

[24] Comita LS, Hubbell SP (2009) Local neighborhood and species' shade tolerance influence survival in a diverse seedling bank. Ecology 90: 328–334. doi:10.1890/08-0451.1. PubMed: 19323215. 19323215

[25] Bartels SF, Chen HYH (2010) Is understory plant species diversity driven by resource quantity or resource heterogeneity? Ecology 91: 1931–1938. doi:10.1890/09-1376.1. PubMed: 20715612. 20715612

[26] Chen HYH, Légaré S, Bergeron Y (2004) Variation of the understory composition and diversity along a gradient of productivity in Populus tremuloides stands of northern British Columbia, Canada. Can J Bot 82: 1314–1323. doi:10.1139/b04-086.

[27] Clark DB, Clark DA, Read JM (1998) Edaphic variation and the mesoscale distribution of tree species in a neotropical rain forest. J Ecol 86: 101–112. doi:10.1046/j.1365-2745.1998.00238.x.

[28] Clark DB, Palmer MW, Clark DA (1999) Edaphic factors and the landscape-scale distributions of tropical rain forest trees. Ecology 80: 2662–2675. Available online at: doi:10.1890/0012-9658(1999)080[2662:EFATLS]2.0.CO;2

[29] Phillips OL, Vargas PN, Monteagudo AL, Cruz AP, Zans MEC et al. (2003) Habitat association among Amazonian tree species: a landscape-scale approach. J Ecol 91: 757–775. doi:10.1046/j.1365-2745.2003.00815.x.

[30] Dormann CF (2006) Effects of incorporating spatial autocorrelation into the analysis of species distribution data. Global Ecol Biogeogr 16: 129–138.

[31] Dormann CF, McPherson JM, Araujo MB, Bivand R, Bolliger J et al. (2007) Methods to account for spatial autocorrelation in the analysis of species distributional data: a review. Ecography 30: 609–628. doi:10.1111/j.2007.0906-7590.05171.x.

[32] Legendre P (1993) Spatial autocorrelation: trouble or new paradigm? Ecology 74: 1659–1673. doi:10.2307/1939924.

[33] Beale CM, Lennon JJ, Yearsley JM, Brewer MJ, Elston DA (2010) Regression analysis of spatial data. Ecol Lett 13: 246–264. doi:10.1111/j.1461-0248.2009.01422.x. PubMed: 20102373. 20102373

[34] Mack RN, Simberloff D, Mark Lonsdale W, Evans H, Clout M et al. (2000) Biotic invasions: causes, epidemiology, global consequences, and control. Ecol Appl 10: 689–710. Available online at: doi:10.1890/1051-0761(2000)010[0689:BICEGC]2.0.CO;2

[35] Brown RL, Peet RK (2003) Diversity and invasibility of southern Appalachian plant communities. Ecology 84: 32–39. Available online at: doi:10.1890/0012-9658(2003)084[0032:DAIOSA]2.0.CO;2

[36] Davies KF, Chesson P, Harrison S, Inouye BD, Melbourne BA et al. (2005) Spatial heterogeneity explains the scale dependence of the native-exotic diversity relationship. Ecology 86: 1602–1610. doi:10.1890/04-1196.

[37] Gilbert B, Lechowicz MJ (2005) Invasibility and abiotic gradients: the positive correlation between native and exotic plant diversity. Ecology 86: 1848–1855. doi:10.1890/04-09997.

[38] Myers N, Mittermeier RA, Mittermeier CG, da Fonseca GAB, Kent J (2000) Biodiversity hotspots for conservation priorities. Nature 403: 853–858. doi:10.1038/35002501. PubMed: 10706275. 10706275

[39] Hart SA, Chen HYH (2008) Fire, logging, and overstory affect understory abundance, diversity, and composition in boreal forest. Ecol Monogr 78: 123–140. doi:10.1890/06-2140.1.

[40] Borcard D, Legendre P, Drapeau P (1992) Partialling out the spatial component of ecological variation. Ecology 73: 1045–1055. doi:10.2307/1940179.

[41] Borcard D, Gillet F, Legendre P (2011) Numerical ecology with R. New York: Springer. 302pp.

[42] Legendre P, Legendre L (1998) Numerical ecology. Amsterdam: Elsevier. 853 pp.

[43] Peres-Neto PR, Legendre P, Dray S, Borcard D (2006) Variation partitioning of species data matrices: estimation and comparison of fractions. Ecology 87: 2614–2625. Available online at: doi:10.1890/0012-9658(2006)87[2614:VPOSDM]2.0.CO;2. PubMed: 17089669 17089669 10.1890/0012-9658(2006)87[2614:vposdm]2.0.co;2

[44] Legendre P, Gallagher ED (2001) Ecologically meaningful transformations for ordination of species data. Oecologia 129: 271–280. doi:10.1007/s004420100716. 28547606

[45] Dray S, Legendre P, Peres-Neto PR (2006) Spatial modelling: a comprehensive framework for principal coordinate analysis of neighbour matrices (PCNM). Ecol Modelling 196: 483–493. doi:10.1016/j.ecolmodel.2006.02.015.

[46] Bellier E, Monestiez P, Durbec JP, Candau JN (2007) Identifying spatial relationships at multiple scales: principal coordinates of neighbour matrices (PCNM) and geostatistical approaches. Ecography 30: 385–399. doi:10.1111/j.0906-7590.2007.04911.x.

[47] Blanchet FG, Legendre P, Borcard D (2008) Forward selection of explanatory variables. Ecology 89: 2623–2632. doi:10.1890/07-0986.1. PubMed: 18831183. 18831183

[48] De'ath G (2002) Multivariate regression trees: a new technique for modeling species-environment relationships. Ecology 83: 1105–1117. doi:10.2307/3071917.

[49] Dufrêne M, Legendre P (1997) Species assemblages and indicator species: the need for a flexible asymmetrical approach. Ecol Monogr 67: 345–366. Available online at: doi:10.1890/0012-9615(1997)067[0345:SAAIST]2.0.CO;2

[50] Anderson MJ, Crist TO, Chase JM, Vellend M, Inouye BD et al. (2011) Navigating the multiple meanings of β diversity: a roadmap for the practicing ecologist. Ecol Lett 14: 19–28. doi:10.1111/j.1461-0248.2010.01552.x. PubMed: 21070562. 21070562

[51] R Development Core Team (2012) R: A language and environment for statistical computing. R Foundation for Statistical Computing, Vienna, Austria.

[52] Tuomisto H, Ruokolainen K, Yli-Halla M (2003) Dispersal, environment, and floristic variation of western Amazonian forests. Science 299: 241–244. doi:10.1126/science.1078037. PubMed: 12522248. 12522248

[53] Tuomisto H, Ruokolainen K, Poulsen AD, Moran RC, Quintana C et al. (2002) Distribution and Diversity of Pteridophytes and Melastomataceae along Edaphic Gradients in Yasuní National Park, Ecuadorian Amazonia1. Biotropica 34: 516–533. Available online at: doi:10.1646/0006-3606(2002)034[0516:DADOPA]2.0.CO;2

[54] Légaré S, Bergeron Y, Paré D (2002) Influence of forest composition on understory cover in boreal mixedwood forests of western Quebec. Silva Fenn 36: 353–366.

[55] Paré D, Bergeron Y (1996) Effect of colonizing tree species on soil nutrient availability in a clay soil of the boreal mixedwood. Can J Forest Res 26: 1022–1031. doi:10.1139/x26-113.

