# Supplementary figures and images for: The Environment, Not Space, Dominantly Structures the Landscape Patterns of the Richness and Composition of the Tropical Understory Vegetation
Source: PLoS One. 2013 Nov 22;8(11):e81308. doi: 10.1371/journal.pone.0081308 (PMC3838366; doi:10.1371/journal.pone.0081308)

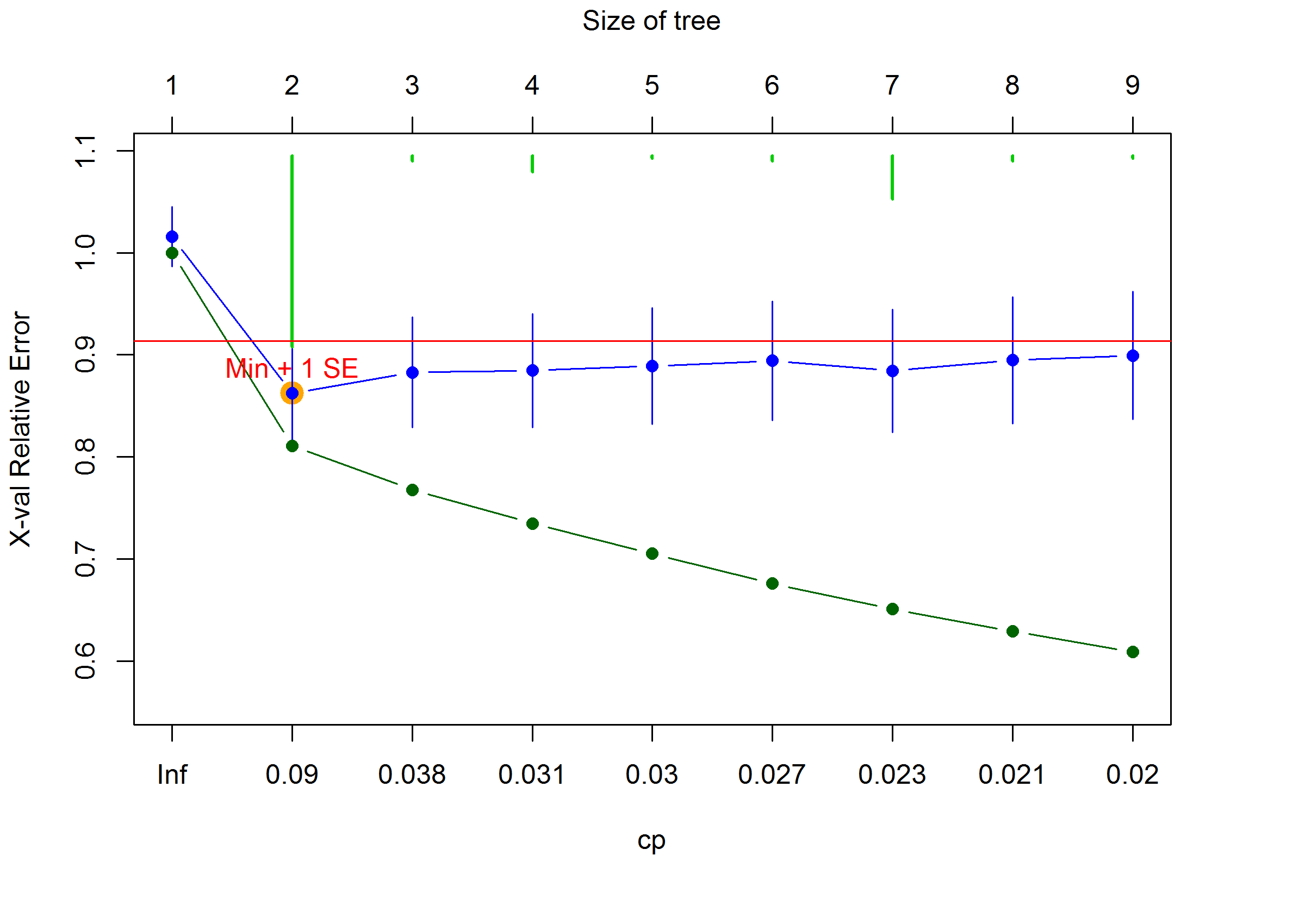

Supplement: Figure S1 — Graph of the relative error and the CVRE for MRT results of all species. The solution with the smallest CVRE is indicated (yellow point), as well as CVRE error bars. The green bars indicate the number of times that the solution was selected as the best during the cross-validation iterations. (TIF) [file pone.0081308.s001.tif]

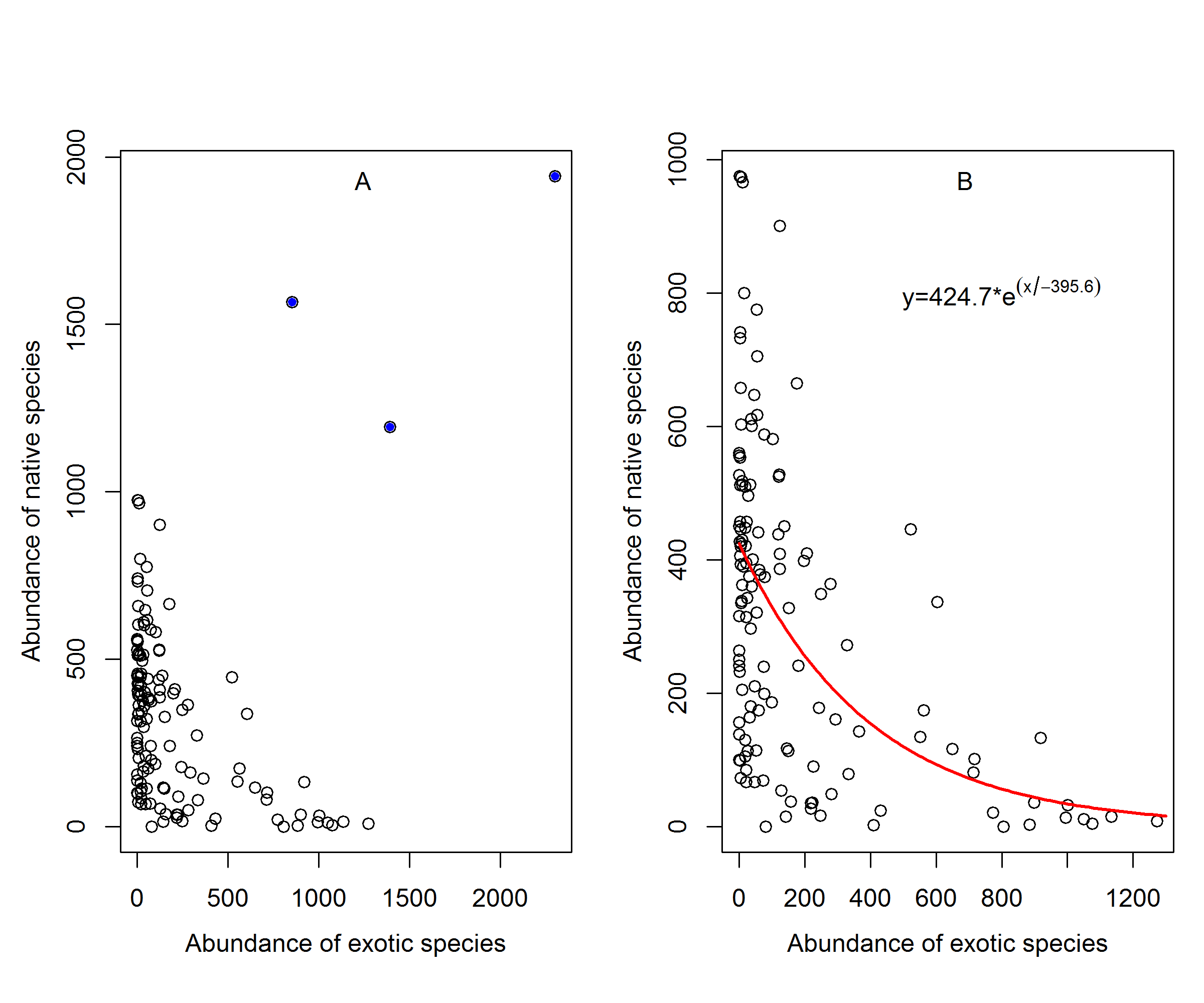

Supplement: Figure S2 — The relationship between the abundances of exotic and native species before and after removing the 3-plot data. The filled blue circles represent the plots that were removed. (TIF) [file pone.0081308.s002.tif]

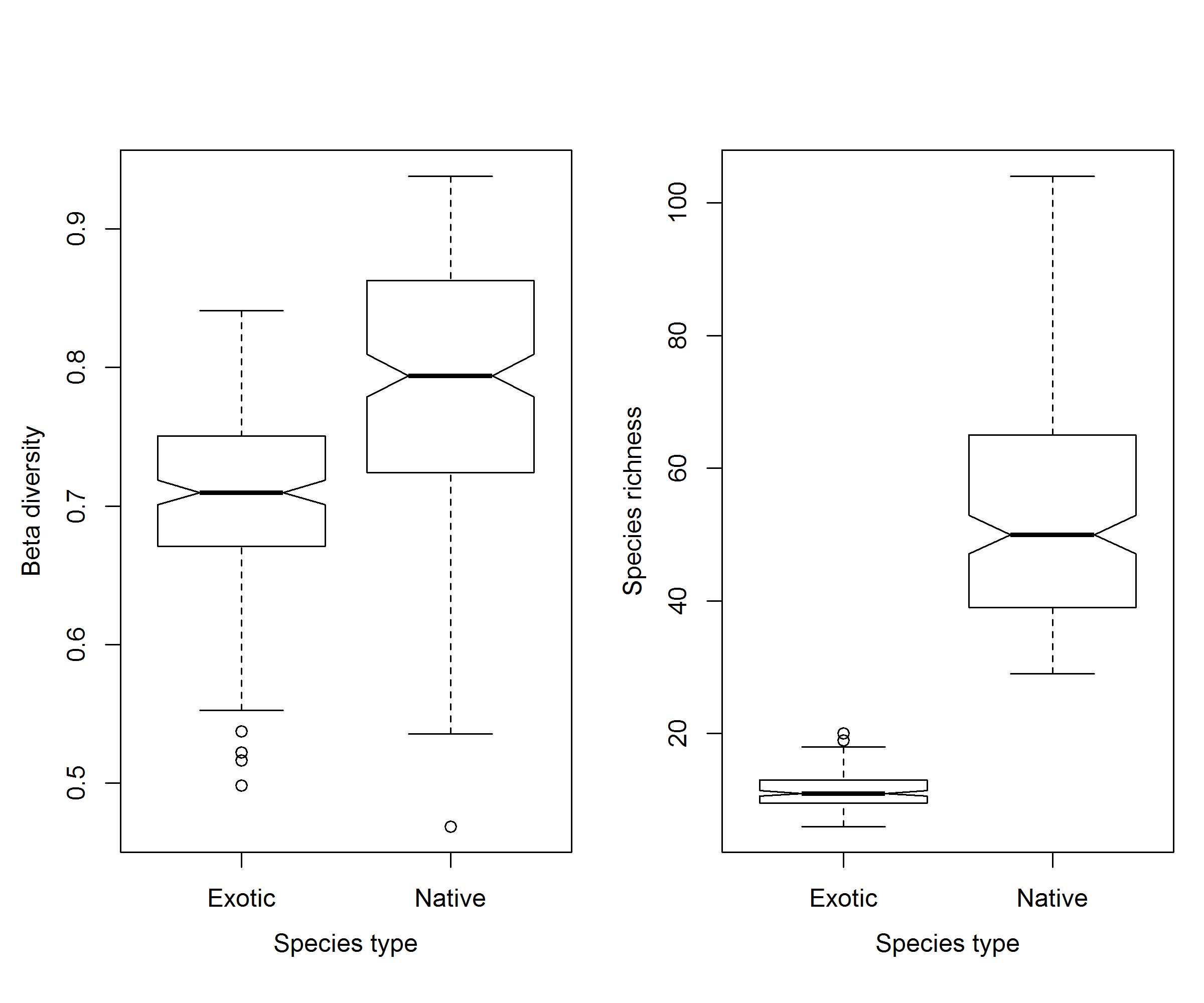

Supplement: Figure S3 — Boxplots of the differences in the beta and alpha diversity values between exotic and native species. (TIF) [file pone.0081308.s003.tif]

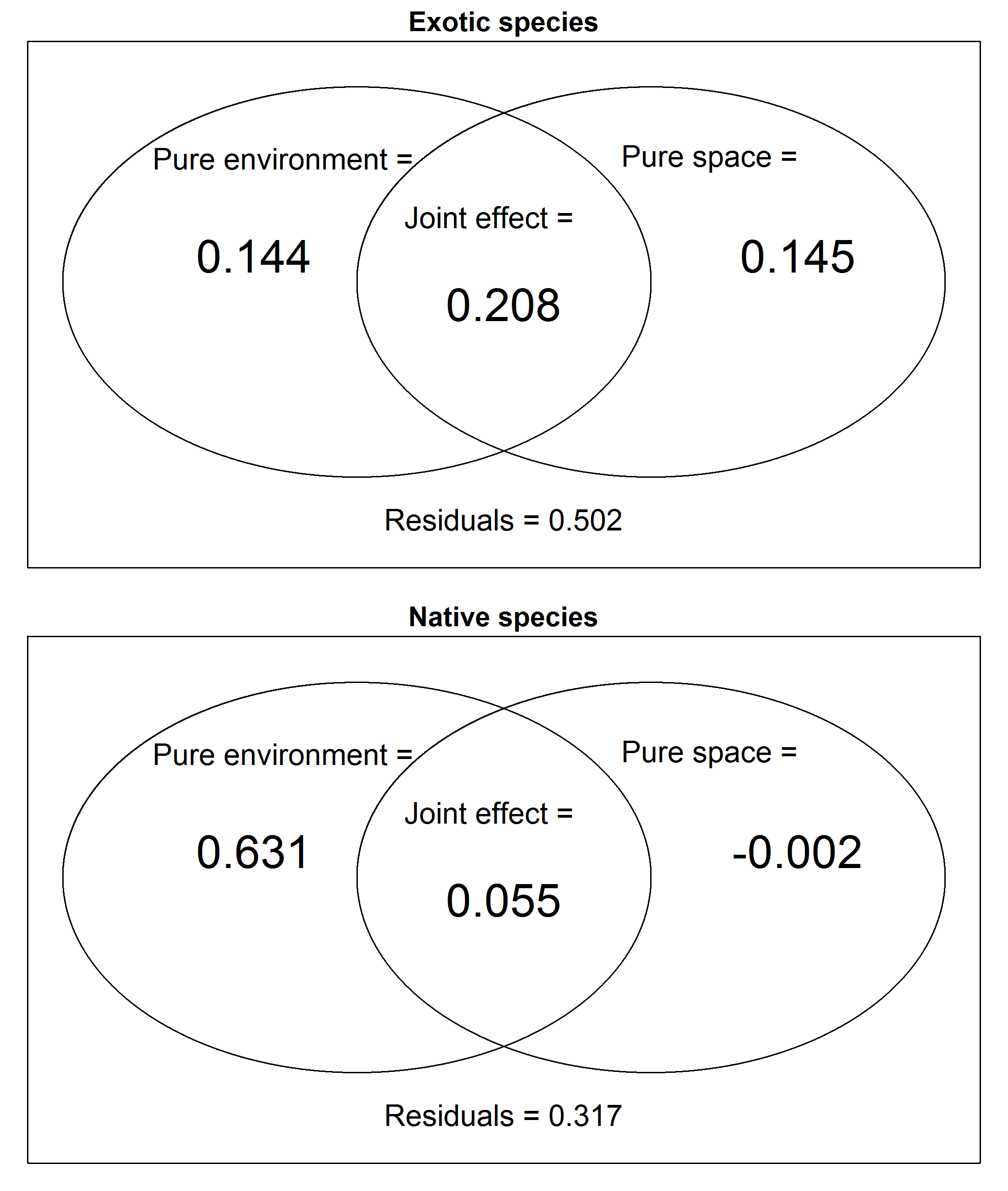

Supplement: Figure S4 — Variation partitioning results of the exotic and native species richness. The two figure panels show Venn diagrams that represent the partitioning of the variations of the richness of the exotic and the native species constrained by the selected environmental variables (environment) and PCNMs (space). The conventions are the same as in Figure 3. (TIF) [file pone.0081308.s004.tif]

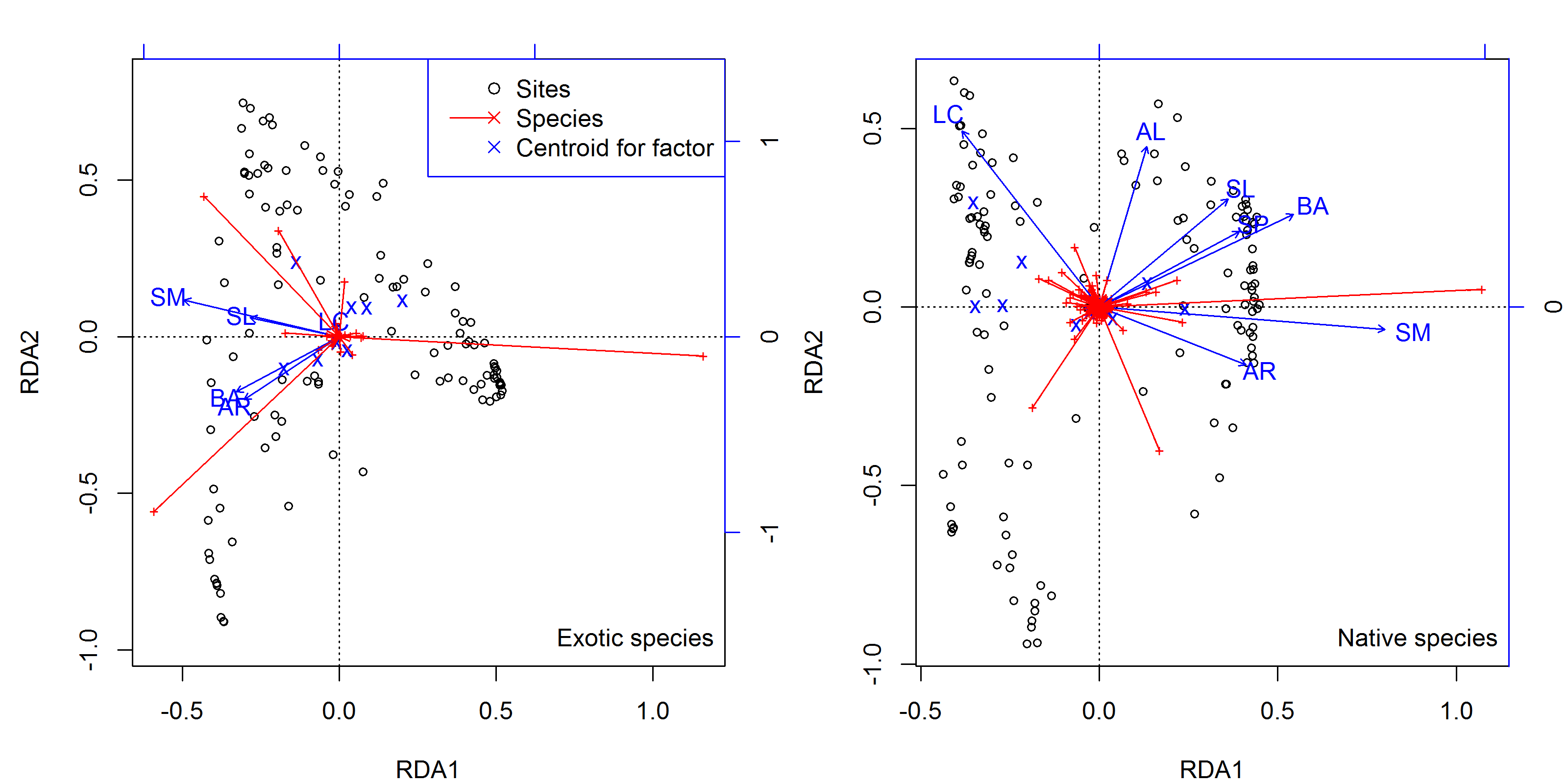

Supplement: Figure S5 — RDA tri-plots of the exotic and native species composition data separately constrained by the selected environmental variables, scaling 2. Abbreviations: AL (altitude), AR (annual rainfall), BA (basal area of trees per square meter), LC (litter coverage), SL (slope), SM (soil moisture), SP (slope position). The bottom and left-hand scales are for the objects and the response variables, the top and right-hand scales are for the explanatory variables. (TIF) [file pone.0081308.s005.tif]

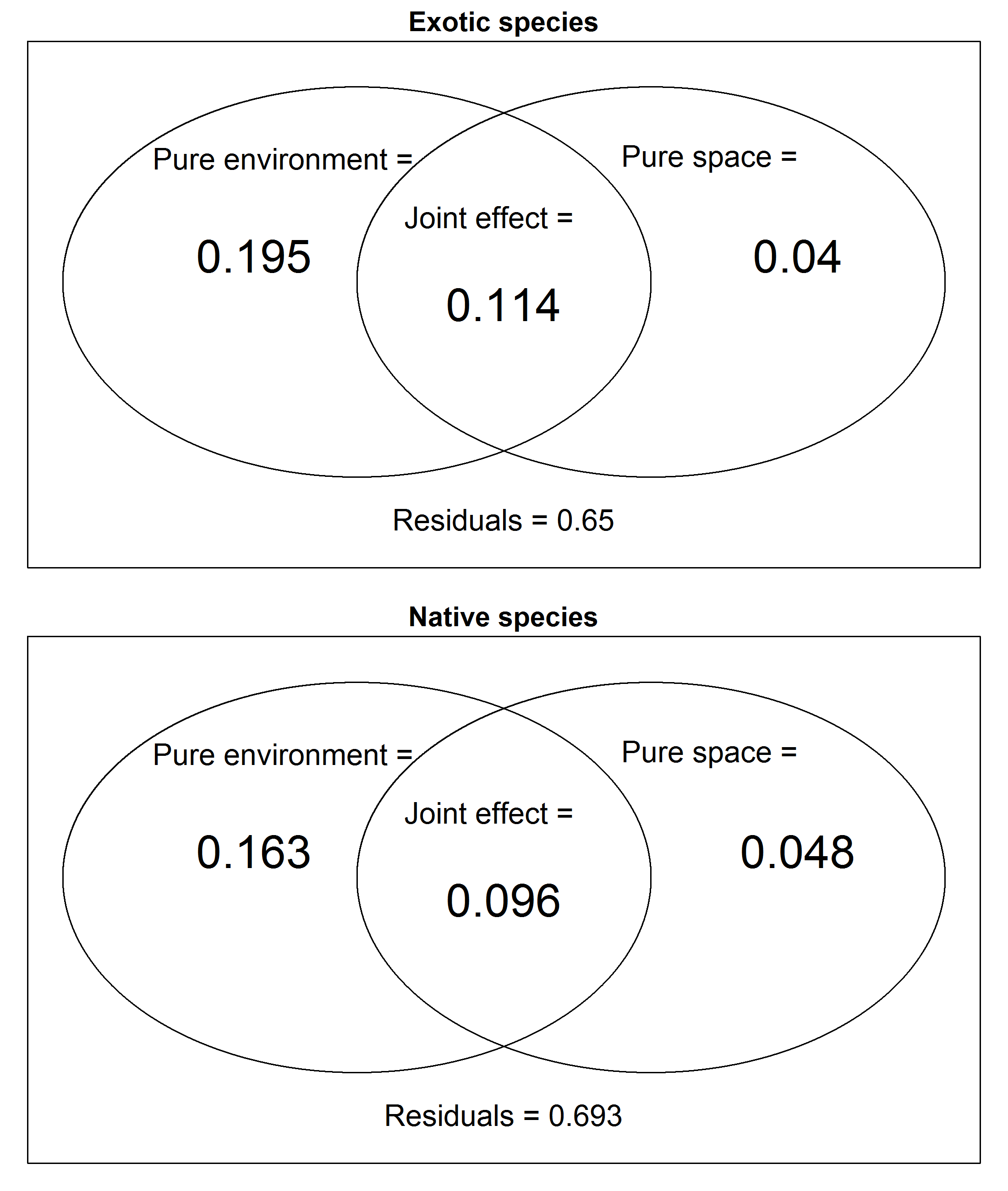

Supplement: Figure S6 — Variation partitioning results of the exotic and native species composition. The two figure panels show Venn diagrams that represent the partitioning of the variations of the exotic and the native species compositions constrained by the selected environmental variables (environment) and PCNMs (space). The conventions are the same as in Figure 3. (TIF) [file pone.0081308.s006.tif]
