# Supplementary material for: The Environment, Not Space, Dominantly Structures the Landscape Patterns of the Richness and Composition of the Tropical Understory Vegetation
Source: PLoS One. 2013 Nov 22;8(11):e81308. doi: 10.1371/journal.pone.0081308 (PMC3838366; doi:10.1371/journal.pone.0081308)
Supplement: Tables S1 — Indicator species for the two MRT groups of all understory plant species. (DOC) [file pone.0081308.s007.doc]

Table S1 Indicator species for the two MRT groups of all understory plant species.

| Species | MRT group | Soil moisture (%) | Indicator value | P-value | Variation partitioned by the MRT tree | Total variation (%) |
| --- | --- | --- | --- | --- | --- | --- |
| *Eupatorium catarium* | 1 | < 11.550 | 0.756 | 0.001 | 8.212 | 18.144 |
| *Rhodomyrtus tomentosa* | 1 | < 11.550 | 0.236 | 0.002 | 0.014 | 0.728 |
| *Erigeron canadensis* | 1 | < 11.550 | 0.174 | 0.022 | 0.002 | 0.067 |
| *Xanthium sibiricum* | 1 | < 11.550 | 0.148 | 0.002 | 0.001 | 0.023 |
| *Corchorus aestuans* | 1 | < 11.550 | 0.130 | 0.003 | 0.004 | 0.308 |
| *Digitaria chrysoblephara* | 1 | < 11.550 | 0.116 | 0.022 | 0.016 | 0.560 |
| *Setaria viridis* | 1 | < 11.550 | 0.105 | 0.021 | 0.017 | 0.964 |
| *Sebastiania chamaelea* | 1 | < 11.550 | 0.093 | 0.007 | 0.000 | 0.003 |
| *Sida acuta* | 1 | < 11.550 | 0.093 | 0.011 | 0.000 | 0.003 |
| *Dactyloctenium aegyptium* | 1 | < 11.550 | 0.093 | 0.007 | 0.041 | 1.173 |
| *Urena procumbens* | 1 | < 11.550 | 0.074 | 0.022 | 0.000 | 0.004 |
| *Chloris virgata* | 1 | < 11.550 | 0.074 | 0.034 | 0.002 | 0.157 |
| *Engelhardia roxburghiana* | 1 | < 11.550 | 0.074 | 0.034 | 0.000 | 0.016 |
| *Abutilon theophrasti* | 1 | < 11.550 | 0.056 | 0.048 | 0.000 | 0.001 |
| *Costus speciosus* | 2 | ≥ 11.550 | 0.088 | 0.047 | 0.000 | 0.018 |
| *Oplismenus compositus* | 2 | ≥ 11.550 | 0.088 | 0.050 | 0.001 | 0.034 |
| *Garcinia oblongifolia* | 2 | ≥ 11.550 | 0.125 | 0.016 | 0.025 | 1.576 |
| *Adiantum capillus-junonis* | 2 | ≥ 11.550 | 0.125 | 0.026 | 0.006 | 0.315 |
| *Lophatherum gracile* | 2 | ≥ 11.550 | 0.143 | 0.036 | 0.008 | 0.381 |
| *Dicranopteris linearis* | 2 | ≥ 11.550 | 0.180 | 0.017 | 0.099 | 4.647 |
| *Blechnum orientale* | 2 | ≥ 11.550 | 0.181 | 0.009 | 0.005 | 0.192 |
| *Lygodium digitatum* | 2 | ≥ 11.550 | 0.213 | 0.003 | 0.001 | 0.039 |
| *Neottopteris antiqua* | 2 | ≥ 11.550 | 0.225 | 0.001 | 0.161 | 3.474 |
| *Pteris cadieri* | 2 | ≥ 11.550 | 0.381 | 0.001 | 0.010 | 0.265 |
| *Axonopus compressus* | 2 | ≥ 11.550 | 0.409 | 0.001 | 0.224 | 2.798 |
| *Ichnanthus vicinus* | 2 | ≥ 11.550 | 0.484 | 0.001 | 0.062 | 0.457 |
| *Acroceras munroanum* | 2 | ≥ 11.550 | 0.721 | 0.001 | 9.646 | 24.890 |
